# Shape-Controlled Growth and *In Situ* Characterization of CdS Nanocrystals via Liquid Cell Transmission Electron Microscopy

**DOI:** 10.3390/molecules29225342

**Published:** 2024-11-13

**Authors:** Wei Wei, Xinyu Sun, Jun Sun, Cen Hao

**Affiliations:** 1 School of Information Technology, Jiangsu Open University, Nanjing 210036, China; apolloxinyusun@126.com (X.S.); sunjun@jsou.edu.cn (J.S.); haocen@jsou.edu.cn (C.H.); 2 Biofuels Institute, School of the Environment, Jiangsu University, Zhenjiang 212013, China

**Keywords:** CdS nanocrystals, *in situ* liquid cell TEM, structural evolution, growth mechanism

## Abstract

Controlling the growth, structure, and shape of CdS nanocrystals is crucial for harnessing their unique physicochemical properties across diverse applications. This control can be achieved by introducing chemical additives into the synthesis reaction mixture. However, precise manipulation of nanocrystal synthesis necessitates a thorough understanding of the formation mechanisms under various chemical conditions, a task that remains challenging. In this study, we employed in situ liquid cell transmission electron microscopy (TEM) to investigate the growth mechanisms of CdS nanocrystals in a reaction solution of cadmium chloride and thiourea, with sodium citrate serving as a structure-directing agent. We observed that CdS nanocrystals evolve through two distinct growth modes: (1) in the absence of sodium citrate, spherical nanocrystals isotropically transform into CdS nanocubes, and (2) in the presence of sodium citrate, cuboid nanocrystals preferentially extend along the {011} direction and anisotropically into CdS triangular nanoplates. Theoretical analysis has confirmed that the adsorption energy of sodium citrate on different crystal facets significantly influences the morphology of the CdS nanocrystals. Our findings not only provide a method for synthesizing CdS nanocrystals based on electron beam induction but also elucidate the intricate nanoscale growth mechanisms, offering insights that could inform the future rational design of nanocrystals with tailored morphologies.

## 1. Introduction

Cadmium Sulfide (CdS) is a significant semiconductor of the II–VI group, known for its direct 2.47 eV band gap at standard room temperature, rendering it a highly efficient optoelectronic material within the ultraviolet–visible spectrum [1,2,3,4], including nanospheroids [5], nanorods [6,7,8], nanowires [9,10], nanocombs [11], and nanobelts [12,13,14]. These nanoparticles, owing to their unique shapes, exhibit distinct dominant facets. Both experimental and computational investigations have highlighted the critical role of these dominant facets’ intrinsic properties in influencing surface reactivity [15], which is crucial for various surface reactions such as adsorption [16,17], oxidation [18], catalysis [19,20,21], and sensing [22,23,24]. During the growth of nanocrystals, the facets with higher energy expand more rapidly than those with lower energy; consequently, the rapid expansion of these high-energy facets leads to their eventual disappearance, stabilizing the nanocrystal with low-energy facets [25,26,27].

Nanotechnology necessitates the synthesis of nanomaterials with specific shapes and sizes tailored for their intended applications [28]. Structure-directing agents represent a distinctive class of ligands or additives that have a profound capability to govern crystal growth, guiding both the shape and size in a controlled manner [29]. These agents are hypothesized to alter the energy of specific crystal facets through preferential adsorption; as a result, this impacts the comparative growth rates of these surfaces and ultimately influences the nanocrystal morphology [30,31,32]. A variety of structure-directing agents have been employed to achieve shape-controlled synthesis of nanomaterials [33]. However, the current models predominantly rely on post-reaction characterizations, leading to limited exploration of the dynamic processes involved in facet growth during nanocrystal synthesis. A more comprehensive understanding of these mechanisms could pave the way for CdS nanocrystals’ innovative strategies to control the shape and structural characteristics.

Recent advancements in the development of liquid cell platforms for in situ transmission electron microscopy (TEM) have made it possible to directly observe and comprehend the nucleation among the nanocrystals’ growth processes from liquid phase precursor solutions with high spatial resolutions [34,35]. Wu et al. investigated the interactions between gold nanoparticles and CdS clusters [36], while Cheng et al. studied the dynamic growth of graphene oxide and CdS nanoclusters in aqueous solutions [37].

Despite these studies, the dynamics of CdS nanocrystal growth remain largely undefined. In the present study, employing a TEM as a carbon film-based liquid cell type, we directly observed the morphological evolution and growth processes of CdS nanocrystals in solution. It was observed that CdS nanocrystals were significantly influenced by sodium citrate in the growth modes. Theoretical calculations have confirmed that the distinct adsorption energies of sodium citrate on different CdS facets were instrumental in dictating the shape-controlled growth mechanisms. Such insights are crucial for the strategic design of nanocrystal facets and the control over functional devices.

## 2. Results and Discussion

**Figure 1:**
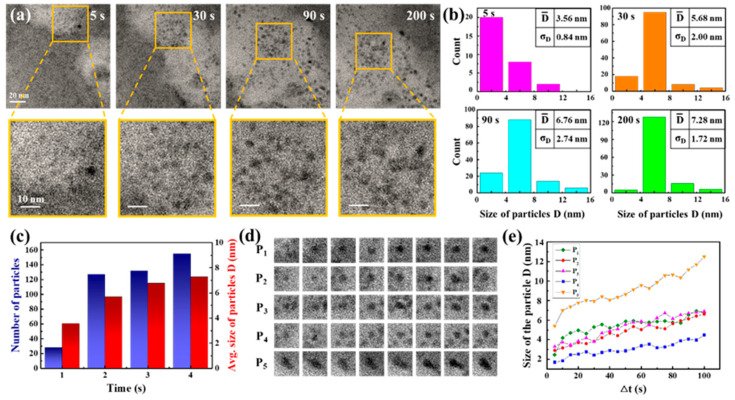
In situ TEM observation of the nanoparticles formed. (**a**) Sequential TEM images captured the primary nanoparticles forming at four time points (5, 30, 90, and 200 s) with a scale bar of 20 nm, along with enlarged images of specific areas with a scale bar of 10 nm. (**b**) Graphs depicted the analysis results for particle size and count at the aforementioned time stamps during particle growth, setting a minimum size limit of 1.5 nm, and (**c**) illustrated changes in total particle number and average sizes based on the results shown in (**b**). (**d**) A series of TEM snapshots tracked the growth of five individual particles labeled as P_1_–P_5_ in solution and (**e**) plotted particle size over time for these five particles.

Carbon film liquid cells, integrated within a high-resolution TEM, facilitated direct in situ observation of nanocrystal growth. The reaction solution was composed of 3 mM cadmium chloride and 9 mM thiourea. A total of 5 μL of ammonia solution was subsequently added to 5 mL of the reaction solution. In Figure 1a, TEM snapshots at four different times (5, 30, 90, and 200 s) from Movie S1 are displayed with magnified images of a specific region, demonstrating the primary nanoparticles’ evolution. Dark and bright regions are shown in the TEM image. Dark regions indicate a thicker liquid layer, and bright regions indicate a thinner liquid layer or bubbles. In order to clarify the patterns of their development, we evaluated the particle size distribution (based on diameter) by tallying and measuring the diameters of particles identified in each frame, as shown in Figure 1b,c. Figure 1b,c show the total number and size of nanoparticles in the TEM images. The TEM images at 60 kx magnification level provided the resolution necessary for detecting larger than 1 nm particles. Observing these particles indicated that the smallest ones, showing growth over time, had initial sizes exceeding 1.5 nm. Our analysis, therefore, focused on quantifying the particle count and sizes, establishing 1.5 nm as the minimum size for consideration. The results indicated a rise in both the mean dimensions and the quantity of particles as time progressed. Specifically, from 28 particles with a 3.56 nm average size at 5 s, the count rose to 155 particles with a 7.28 nm average size through 200 s. Snapshot sequences in Figure 1d illustrate the development of five specific particles over 100 s, and Figure 1e plots the size changes in these particles over time, showing initial sizes of approximately 3 nm, which increased to about 6 nm within 100 s. The growth in particle size may be attributed to the process of atomic addition based on traditional growth theories or self-catalyzed surface expansion. This involves metal ions adhering to the surface of the particle, undergoing reduction, and integrating into the surface atoms. The rapid growth observed in these primary particles suggests enhanced surface interactions for the nanoparticles. Given the composition of the reaction solution and the conditions under which the reaction occurred, it is speculated that the resulting nanoparticles are CdS nanocrystals.

**Figure 2:**
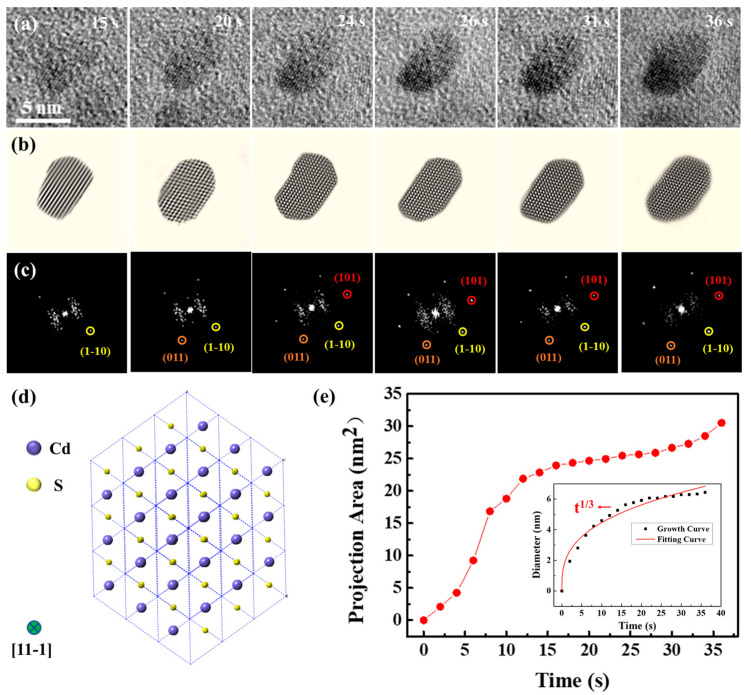
High-resolution images capturing the growth of a solitary CdS nanocrystal from a movie. (**a**) Monomer growth of the CdS nanocrystal. (**b**) Sequentially displayed filtered images and (**c**) corresponding FFT images, highlighting a single CdS nanocrystal developed. (**d**) Two-dimensional projection of CdS along the {11-1} view zone axis. The green circle represents the view zone axis. (**e**) Change in the projection area of the CdS nanocrystal with time. The illustration shows the change in the effective diameter of the CdS nanocrystal.

To determine the composition of the nanoparticles, atomic-resolution snapshots of a single nanocrystal’s growth process were captured (Figure 2 and Movie S2). The experimental data in Figure 2 were obtained from the same liquid cell in Figure 1. The magnification of Movie S2 was 250 kx. Examination of high-resolution TEM images and corresponding FFT images (Figure 2a–c) confirmed that the nanocrystal was consistent with a CdS structure, which was further verified as CdS (JCPDS No. 43-0985). In order to clarify the growth mechanisms of CdS nanocrystals, an analysis was conducted on the chemical composition of the initial solution. Interactions occur when the electron beam traverses a liquid pocket, enabling the interaction of liquid molecules with the beam, resulting in the formation of intermediate species, including O_2_, H_2_, H⁺, OH⁻, H_2_O_2_, H_3_O⁺, and e⁻_aq_, in concentrations that are dependent on dose rate, total dose, and irradiation pattern [38]. The latest study by Fritsch et al. can assess the magnitude of beam-induced radiolysis through radiation chemical simulations [39,40,41]. Their results indicate that sparse kinetic models can accurately describe steady-state formation during liquid-phase TEM and provide a handy prerequisite for efficient multidimensional modeling. These interactions facilitate a series of growth processes [42], depicted through the chemical reactions below:

(1) $${Cd}^{2+}+{SC\left(NH_{2}\right)}_{2}+2OH^{-}\to CdS+CN_{2}H_{2}+2H_{2}O$$

High-resolution TEM pictures and their respective FFT images elucidated structural changes in a CdS nanocrystal during its growth. The CdS nanocrystal exhibited growth along the {1-10}, {011}, and {101} directions. By 36 s, the nanocrystal had evolved into an ellipsoidal shape and reached an equilibrium state. Notably, the FFT images revealed that the CdS nanocrystal could undergo slight rotational movements within the solution. Predominantly, the CdS nanocrystal was observed along the {11-1} axis, with its two-dimensional structure diagram presented in Figure 2d. Figure 2e depicts the growth kinetics of the nanocrystals, highlighting a pivotal moment in the growth kinetics curve at approximately 18 s. Initially, the CdS nanocrystal experienced rapid growth, which subsequently transitioned to a slower rate post-18 s. At 36 s, the projection area of the CdS nanocrystal had expanded to 30.55 nm^2^. Generally, nanocrystals exhibit faster growth rates at the onset, which is a common phenomenon among most nanocrystals [43,44]. To further explore the growth dynamics of the CdS nanocrystal, changes in size over time were quantitatively analyzed, with results illustrated in Figure 2e. The CdS nanocrystal approximated a spherical shape, and its size was expressed through the effective diameter, calculated using the formula *d =* 2 *× √(A⁄π)*, where A represents the projected area of the nanocrystal. The classical Lifshitz–Slyozov–Wagner (LSW) theoretical model for growth dynamics served as the quantitative analytical framework [45,46,47]. According to the LSW model, when the size-time relationship of the nanocrystals follows *D* ∝ *t*^1⁄3^, the growth is diffusion-limited. Conversely, when the relationship follows *D* ∝ *t*^1⁄2^, it indicates reaction-limited growth. Data fitting indicated that the size evolution of the CdS nanocrystal during growth adhered approximately to *t*^1⁄3^, verifying that the growth in solution was diffusion-limited.

**Figure 3:**
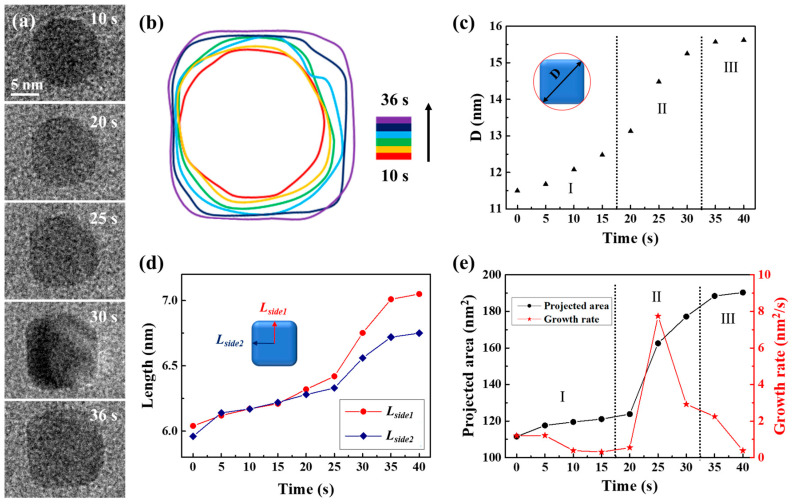
Quantitative analysis of CdS nanocube’s growth process in a carbon film liquid cell. (**a**) TEM image sequences show shape transformations from a sphere into a CdS nanocube. (**b**) The corresponding time-labeled contours illustrate the formation process. (**c**) Variations in the diameter of the circumscribed circle of the nanocube correspond to its growth at different stages of the reaction. The Roman numerals and dashed lines are used to divide the different growth stages in the diagram. (**d**) The size development as *L_side_*_1_ and *L_side_*_2_ represent two distances from the center to the side of the nanocube. (**e**) Plots of the projected area and growth rate of CdS nanocube with time.

Using the same precursor solution, the liquid cell was fabricated again to observe the transformation of spherical nanocrystals into CdS nanocubes. Time-lapse images were captured to document the process of a single CdS nanocube undergoing shape transformation in a precursor solution (3 mM cadmium chloride and 9 mM thiourea), as illustrated in Figure 3a. The complete development of the morphological evolution of the CdS nanocrystal was recorded in Movie S3. The magnification was 250 kx. After 36 s, the primary spherical nanocrystal developed into a CdS nanocube. The transition from sphere to cube was detailed in a time-domain contour plot as Figure 3b displayed, which depicted the evolution of the nanocrystal’s shape through changes in its contour plots during the cube formation. As the changes quantitatively described in shape and size of the CdS nanocrystal, we observed how the size of an individual CdS nanocrystal changed over time from Figure 3c–e. We used the diameter of the circumscribed circle (*D*) as a measure to track both shape and size evolution, as shown in Figure 3c. The process of shape transformation was divided into three distinct phases. In the initial phase (Stage I), *D* consistently increased with reaction time due to the formation of new uniform layers through the attachment of CdS monomers. During the second stage (Stage II), the diameter (*D*) increased rapidly as the shape of the CdS nanocrystal transitioned from a sphere to a nanocube. In the third stage (Stage III), the increase in *D* slowed due to the gradual shaping of the nanocube, resulting in the preservation of a consistent structure. In order to better understand the morphological evolution mechanism of the CdS nanocube during its growth process, we examined the variations in two distances from the center of the CdS nanocrystal to its edges (*L_side_*_1_ and *L_side_*_2_), as shown in Figure 3d. The data suggest a consistent increase in the values of *L_side_*_1_ and *L_side_*_2_ as the 0 to 35-s reaction time progresses. Notably, the growth rates and trends of *L_side_*_1_ and *L_side_*_2_ were essentially identical, suggesting simultaneous growth of the exposed facets. As Figure 3e depicted, the CdS nanocrystal projected area initially grew slowly, then accelerated, and eventually stabilized. Specifically, the growth rate correlated with the statistical results in Figure 3c,d, with a significant increase observed during the shape transformation (Stage II). Through this quantitative analysis of the CdS nanocube’s growth process, we identified two significant phenomena: firstly, the growth rate of the CdS nanocrystal increased markedly during the transition from sphere to nanocube; secondly, the growth rates of the exposed facets were essentially uniform, indicating that the growth of the CdS nanocube was an isotropic process.

**Figure 4:**
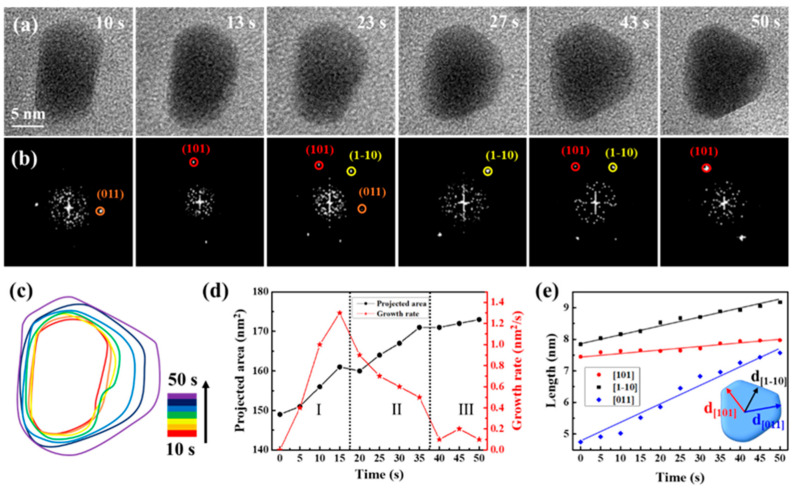
Quantitative analysis of CdS triangular nanoplate’s growth process following sodium citrate added to the precursor solution. (**a**) Time-lapse TEM images depict the growth process. (**b**) Corresponding FFT images highlight the structural evolution. (**c**) Time-labeled contours demonstrate the formation process. (**d**) Graphs display projected area and growth rate over time. The Roman numerals and dashed lines are used to divide the different growth stages. (**e**) The measured average distances from the nanocrystal center to (101), (1-10), and (011) facets are presented as a time function.

Serving as a structure-controlling agent, sodium citrate is crucial in methodically controlling the formation of nanocrystals and refining growth conditions [42,48,49,50]. Upon introducing sodium citrate to the precursor solution in the remanufactured liquid cell, different growth modes of CdS nanocrystals were observed, as depicted in Figure 4, sourced from Movie S4. The magnification of Movie S4 was 250 kx. Figure 4a,b display sequential images that illustrate the growth process and structural evolution of a CdS nanocrystal, primarily oriented along the {11-1} axis. During the initial growth stage (0 to 50 s), the three low-energy facets ((101), (1-10), and (011)) were identifiable, with the nanocrystal exhibiting frequent, slight rotations. The shape of the CdS nanocrystals evolved from cuboids to triangular nanoplates, suggesting preferential growth along specific directions. The time-domain contour plot corresponded, as Figure 4c depicted, further details the shape evolution of the CdS nanocrystals. Figure 4d illustrates that the projected area of the CdS nanocrystals continued to grow. However, the growth rate initially increased, then rapidly decreased, and finally stabilized. Notably, during the shape transformation process (Stage II), the growth rate of the CdS nanocrystal significantly decreased from 1.3 nm/s to 0.5 nm/s, reflecting the cessation or slowdown of growth in some exposed facets, while the shape transformation was significantly influenced. We meticulously analyzed the crystal facets’ evolution through the measurement of a CdS nanocrystal’s changing shape and the tracking of various facets’ progress. In Figure 4e, we have illustrated the average distances from the center of the nanocrystal to the (101), (1-10), and (011) facets over time. The growth rates along these three directions varied distinctly; within 50 s, the CdS nanocrystal grew marginally from 7.45 nm to 7.97 nm along {101}, yet exhibited significant growth along {1-10} and {011}, particularly along {011}, where it grew most notably and fastest by 3 nm. Consequently, the presence of sodium citrate led to anisotropic growth, with CdS nanocrystals preferentially expanding along {011}, facilitating the transformation from cuboids to triangular nanoplates. We hypothesize that the anisotropic growth pattern of the CdS nanocrystals was due to differential adsorption of sodium citrate on the exposed facets.

**Figure 5:**
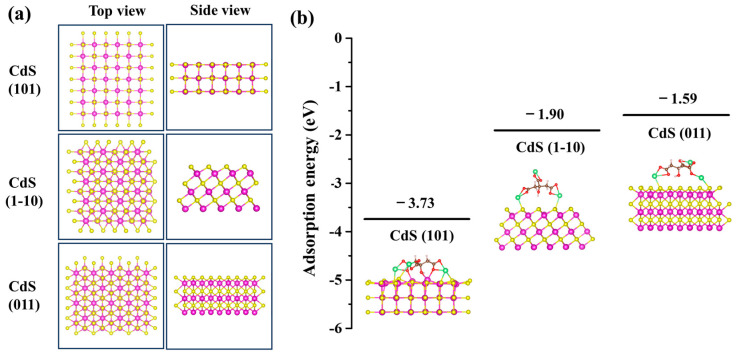
DFT simulations of sodium citrate adsorbed on CdS (101), (1-10) and (011) facets. (**a**) Configurations of CdS (101), (1-10), and (011) facets. (**b**) Adsorption energies of sodium citrate adsorbed on CdS (101), (1-10) and (011) facets. The illustrations show the DFT-calculated adsorption configuration of sodium citrate on CdS facets. Atoms are noted with colors (purple: cadmium; yellow: sulfur; brown: carbon; red: oxygen; pink: hydrogen; green: sodium).

To confirm our hypothesis regarding the selective preference for specific crystal facets induced by sodium citrate, we employed DFT to calculate the adsorption energies and configurations of sodium citrate on the CdS (101), (1-10), and (011) facets. Figure 5a illustrates the configurations of these CdS facets. During the optimization process, the atoms in the bottom two layers of the CdS (101) and the bottom three layers of the CdS (1-10) and (011) were fixed, while all other atoms were allowed to relax in all DFT calculations. The calculated adsorption energies indicated significant differences (Figure 5b), with a negative adsorption energy denoting a stable adsorption structure. Specifically, the adsorption energies for sodium citrate on the CdS (101), (1-10), and (011) facets were −3.73 eV, −1.90 eV, and −1.59 eV values, respectively. This reveals a strong interaction between sodium citrate and the CdS facets, particularly showing that sodium citrate can be stably adsorbed on these facets. Notably, sodium citrate exhibited the highest adsorption energy on the CdS (101) facet, suggesting a stronger affinity for this facet, which consequently limits its growth, causing the CdS nanocrystal to grow most slowly along the {101} direction. This observation aligns with our experimental findings. Conversely, the CdS (011) facet displayed the lowest adsorption energy, implying that the presence of sodium citrate least affects its growth. Hence, CdS nanocrystals exhibit preferential growth along the {011} direction. Thus, we conclude that the adsorption energy of ligands at different crystal facets is a critical parameter in controlling shape during the growth of CdS nanocrystals.

## 3. Materials and Methods

### 3.1. Sample Preparation

Cadmium chloride, ammonia solution (NH_3_·H_2_O, 28%), thiourea, and sodium citrate were procured from Aladdin and used as is. The CdS nanocrystal synthesis was composed of two distinct mixtures as the precursor solutions: (i) a 3 mM solution of cadmium chloride combined with a 9 mM solution of thiourea; (ii) a 3 mM solution of cadmium chloride, a 9 mM solution of thiourea, and a 0.05 mM solution of sodium citrate. To each 5 mL of these precursor solutions, 5 μL of ammonia solution was subsequently added.

### 3.2. The Fabrication of Liquid Cell

A total of 2.5 μL reaction solution was sandwiched between two TEM copper grids to create a slender liquid layer enclosed by carbon films. The liquid cell was allowed to dry under ambient conditions for 3 h. Liquid pockets may remain sealed between the carbon films due to van der Waals forces, thereby preserving them for subsequent TEM analysis. The volume of the liquid pockets was about 10^5^ nm^3^. Figure S7 shows the TEM images of liquid pockets.

### 3.3. In Situ TEM Observation and Experiment

The in situ growth of CdS nanocrystals was observed in real-time using a JEOL 2010 FEG TEM. The electron beam passed through the carbon film, which facilitated the growth of the CdS nanocrystals. The beam dose rate is 3.2 × 10^8^ Gy/s under a magnification of 60 kx. When the magnification is increased to 250 kx, the beam dose rate is 5.7 × 10^9^ Gy/s. Dynamic sequences were captured at a rate of one frame per second.

## 4. Conclusions

In conclusion, we have successfully utilized in situ liquid cell TEM to directly visualize the unique CdS nanocrystals’ shape transformation processes. The observations indicated that sodium citrate has a significant impact on these nanocrystals’ growth modes. We identified two separate growth modes: in the absence of sodium citrate, CdS nanocubes exhibited isotropic growth, and the growth rate of the nanocrystals significantly increased during the transformation from spherical to cubic forms. Conversely, when sodium citrate is present, the growth of CdS triangular nanoplates exhibited anisotropic growth, favoring expansion along the {011} direction. During this transformation from cuboids to triangular nanoplates, the growth rate of the CdS nanocrystals decreased markedly, with some exposed facets ceasing growth or growing at a reduced pace. Theoretical analysis has confirmed that the adsorption energy of sodium citrate on different crystal facets ((101) > (1-10) > (011)) significantly influences the morphological development of the CdS nanocrystals. The addition of sodium citrate exerted the least effect on the CdS (011) facets, leading to preferential growth along this orientation. These findings improve our understanding of the transitional substances involved in the conversion processes of various nanomaterials and underscore the impact of molecular adsorption on crystal growth dynamics. Moreover, this study not only enhances our comprehension of the mechanisms involved in synthesizing CdS nanocrystals but also demonstrates how a blend of experimental and theoretical approaches can be employed to rationally engineer controllable nanomaterials. These insights are instrumental in developing strategies for synthesizing nanomaterials with desired properties and configurations.

## Data Availability

The original contributions presented in the study are included in the article and Supplementary Materials.

## Supplementary Materials

The following supporting information can be downloaded at: https://www.mdpi.com/article/10.3390/molecules29225342/s1, Table S1: DFT calculations to determine the adsorption energies of sodium citrate adsorbed on CdS (101), (1-10) and (011) facets. Table S2: Steady state concentration of radiolysis products at dose rate of 5.7 × 10^9^ (Gy/s). Figure S1: Preparation of a carbon film-based liquid cell. Figure S2: Quantitative analysis of the growth process of CdS nanocube in a carbon film liquid cell. Figure S3: Quantitative analysis of the growth process of CdS triangular nanoplate after adding sodium citrate to the precursor solution. Figure S4: DFT-calculated configuration of CdS (101) surface and the adsorption configuration of sodium citrate on CdS (101) surface. Figure S5: DFT-calculated configuration of CdS (1-10) surface and the adsorption configuration of sodium citrate on CdS (1-10) surface. Figure S6: DFT-calculated configuration of CdS (011) surface and the adsorption configuration of sodium citrate on CdS (011) surface. Figure S7: TEM images of liquid pockets. Figure S8: (a) A series of TEM snapshots tracked the growth of three individual nanoparticles labeled as P_1_–P_3_ in solution. (b) The change in the effective diameter of the CdS nanocrystal. Movie S1: In situ TEM movie shows the formation process of CdS nanoparticles in precursor solution corresponding to Figure 1. Movie S2: In situ TEM movie shows the growth process of a single CdS nanocrystal with an atomic resolution corresponding to Figure 2. Movie S3: In situ TEM movie shows the growth process of the CdS nanocube without the addition of sodium citrate, corresponding to Figure 3. Movie S4: In situ TEM movie shows the growth process of CdS triangular nanoplate in the presence of sodium citrate, corresponding to Figure 4.
