# Supplementary material for: Shape-Controlled Growth and In Situ Characterization of CdS Nanocrystals via Liquid Cell Transmission Electron Microscopy
Source: Molecules. 2024 Nov 13;29(22):5342. doi: 10.3390/molecules29225342 (PMC11596350; doi:10.3390/molecules29225342)
Supplement: Supplementary file 1 [file molecules-29-05342-s001.zip › molecules-3275868-supplementary.pdf]

Supporting Information

Shape-controlled growth and *in situ* characterization of CdS nanocrystals via liquid cell transmission electron microscopy

Wei Wei, * Xinyu Sun, Jun Sun, and Cen Hao

1 Density Functional Theory (DFT)

1.1. Computational Models and Methods

All density functional theory (DFT) calculations were carried out in the the CP2K code. All calculations employed a mixed Gaussian and planewave basis sets. Core electrons were represented with norm-conserving Goedecker-Teter-Hutter pseudopotentials, and the valence electron wavefunction was expanded in a double-zeta basis set with polarization functions along with an auxiliary plane wave basis set with an energy cutoff of 400 eV. The generalized gradient approximation exchange-correlation functional of Perdew, Burke, and Enzerhof (PBE) was used. Each configuration was optimized with the Broyden-Fletcher-Goldfarb-Shanno (BGFS) algorithm with SCF convergence criteria of 1.0×10^{-6} au. The van der Waals correction of Grimme's DFT-D3 model was also adopted. The CdS (101) surface was model as a p (3 × 3) supercell slab with three layers which comprises of 54 Cd and 54 S atoms. The CdS (1-10) surface was model as a p (2 × 4) supercell slab with six layers which comprises of 48 Cd and 48 S atoms. The CdS (011) surface was model as a p (4 × 2) supercell slab with six layers which comprises of 48 Cd and 48 S atoms. Then, a vacuum height of 15 Å in the Z direction was inserted between the periodic CdS (101), (1-10) and (011) slabs to eliminate any unphysical interaction. During the optimization, the atoms in the bottom two layer of the CdS (101) and the bottom three layer of the CdS (1-10) and (011) slabs were frozen while all other atoms were relaxed in all DFT calculations.

1.2 Adsorption Energy

The adsorption energy between the adsorbate and the CdS surface substrate can be calculated

using the following equation:

$$\Delta E_{ads} = E_{adsorbate@\text{substrate}} - E_{\text{substrate}} - E_{adsorbate} \quad \text{(S1)}$$

In Eq. (S1), $E_{adsorbate@\text{substrate}}$ and $E_{\text{substrate}}$ represent the total energies of the CdS substrate with and without the adsorption, respectively. $E_{adsorbate}$ is the total energy of the adsorbate. According to this equation, a negative adsorption energy corresponds to a stable adsorption structure (Table S1).

Table S1. DFT calculations to determine the adsorption energies of sodium citrate adsorbed on CdS (101), (1-10) and (011) facets.

Configuration	E (A.U)	△E (eV)
Sodium-citrate	-291.06	
CdS (101)	-3041.43	
Sodium-citrate @ CdS (101)	-3332.62	-3.73
CdS (1-10)	-2702.53	
Sodium-citrate @ CdS (1-10)	-2993.66	-1.90
CdS (011)	-2702.53	
Sodium-citrate @ CdS (011)	-2993.65	-1.59

2. Electron dose and radiolysis product estimation

Gray (Gy) which is defined as the adsorption of one joule of energy per kilogram of water, is used as the unit for dose to describe the radiation effect of incident electrons upon the thin liquid film. When the liquid layer thickness is on the order of electron's mean free path or smaller, the dose rate Φ can be calculated by equation (J Phys Chem C, 2014, 118: 22373-22382):

$$\emptyset = \frac{10^5 SI}{\pi a^2} \text{ (Gy/s)}$$

Here S (MeV electron cm^2/g) represents the stopping power in water, I (C/s) is the electron beam current and a (m) is the beam radius. Electron stopping power of water is adopted from the ESTAR database available from NIST (NIST Stopping-Power and Range Tables: Electrons, Protons, Helium Ions. https://physics.nist.gov/PhysRefData/Star/Text/ESTAR.html). The beam dose rate (360 $e^- Å^{-2} s^{-1}$) in our experiment is calculated as 5.7×10^9 (Gy/s). The corresponding steady state concentration of radiolysis products can be approximated with a power law (J Phys Chem C, 2014, 118: 22373-22382):

$$C_{SS,i} \sim \alpha_i \emptyset^{\beta_i}$$

We calculated the concentration of several products at the dose rate of 5.7×10^9 (Gy/s), as shown in Table S2. The parameters α_i and β_i are obtained from the reference.

Table S2. Steady state concentration of radiolysis products at dose rate of 5.7×10^9 (Gy/s).

	Steady State Concentration (mM) at dose rate of 5.7×10^9 (Gy/s)
H_2O_2	5.7
OH^-	0.58
HO_2•	0.23
O_2	0.85
H•	0.044

3. Supporting Movies

Movie S1: *In-situ* TEM movie (accelerated 10 times) shows the formation process of CdS nanoparticles in precursor solution corresponding to Figure 1.

Movie S2: *In-situ* TEM movie (accelerated 2 times) shows the growth process of a single CdS nanocrystal with atomic-resolution corresponding to Figure 2.

Movie S3: *In-situ* TEM movie (accelerated 3 times) shows the growth process of CdS nanocube without the addition of sodium citrate corresponding to Figure 3.

Movie S4: *In-situ* TEM movie (accelerated 3 times) shows the growth process of CdS triangular nanoplate in the presence of sodium citrate corresponding to Figure 4.

4. Supporting Figures

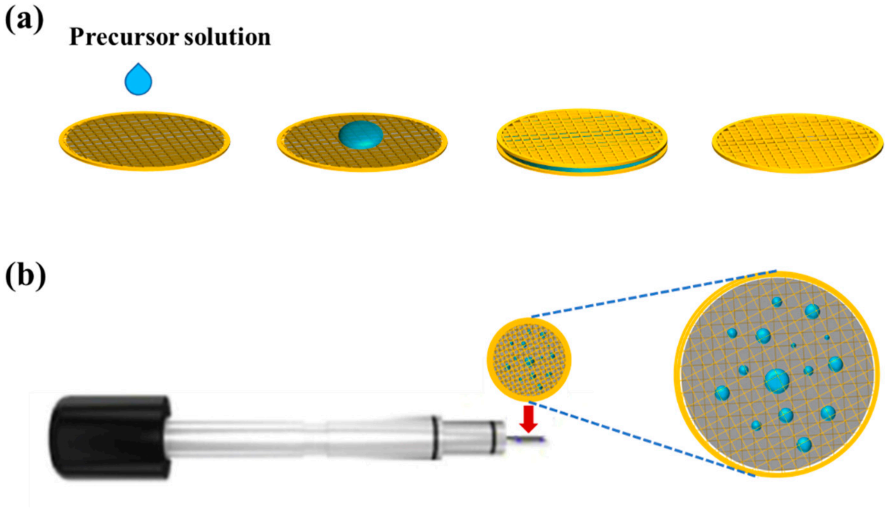

Figure S1. Preparation of a carbon film liquid cell.

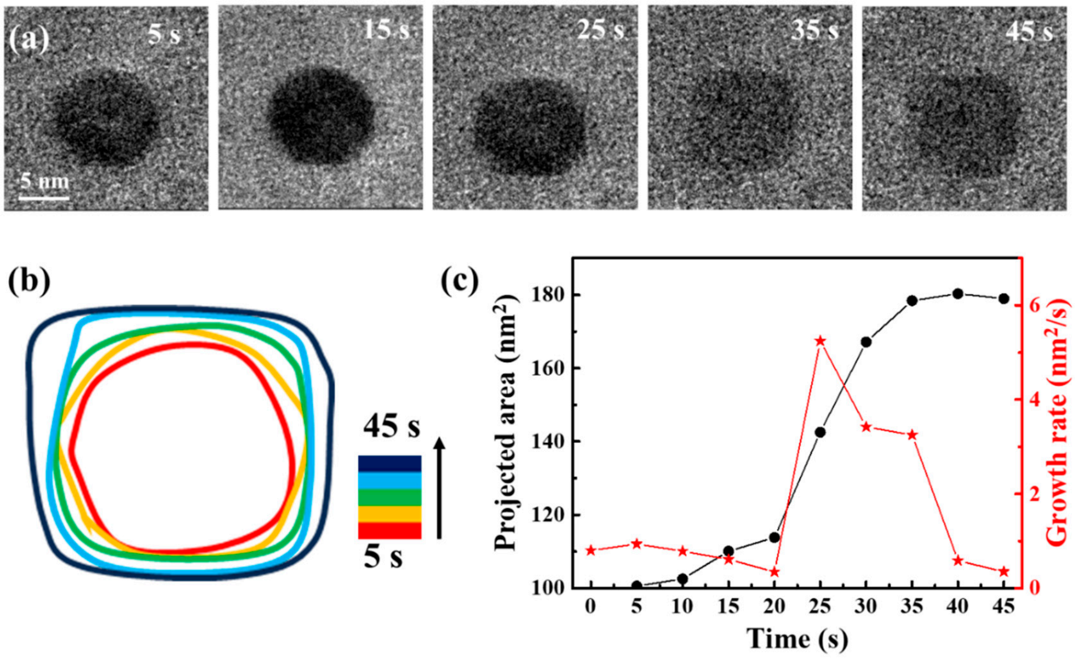

Figure S2. Quantitative analysis growth process of CdS nanocube in a liquid cell. (a) TEM image sequences show shape transformations from sphere into CdS nanocube. (b) The

corresponding time-labeled contours show the formation process of the CdS nanocube. (c) Plots of the projected area and growth rate of CdS nanocube with time.

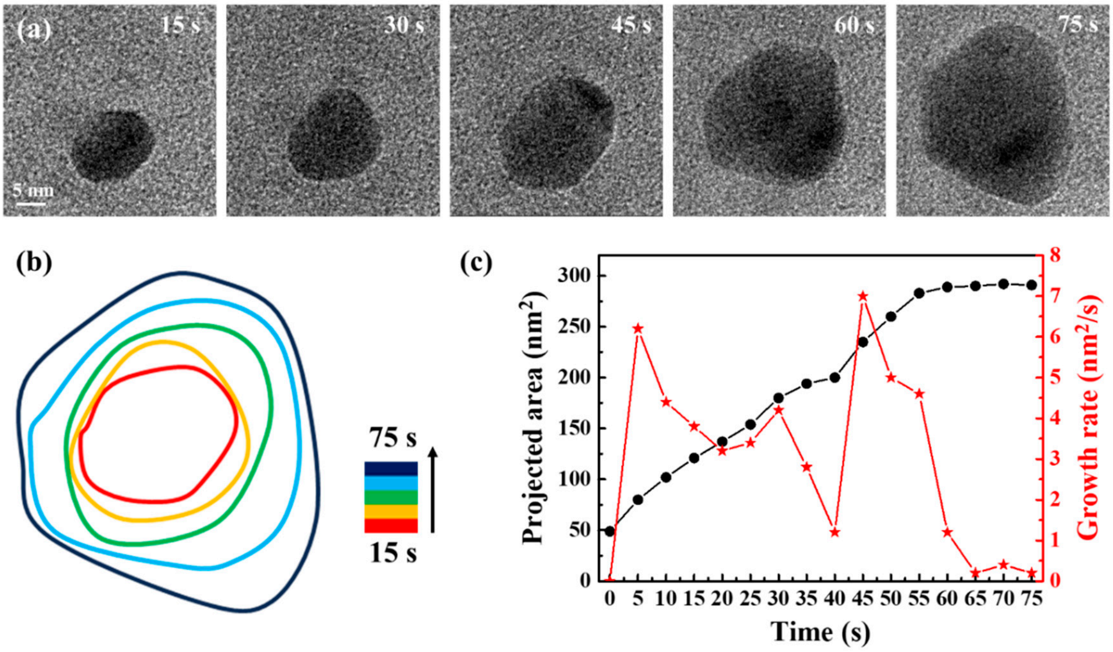

Figure S3. Quantitative analysis growth process of CdS triangular nanoplate after adding sodium citrate to the precursor solution. (a) Time-lapse TEM images show the growth process of CdS triangular nanoplate. (b) The corresponding time-labeled contours show the formation process of the CdS triangular nanoplate. (c) Plots of the projected area and growth rate of CdS triangular nanoplate with time.

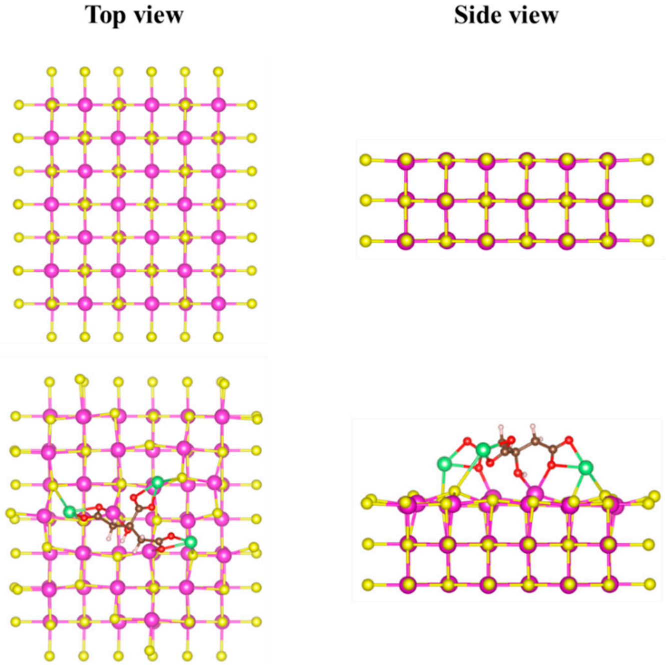

Figure S4. DFT-calculated configuration of CdS (101) surface and the adsorption configuration of sodium citrate on CdS (101) surface.

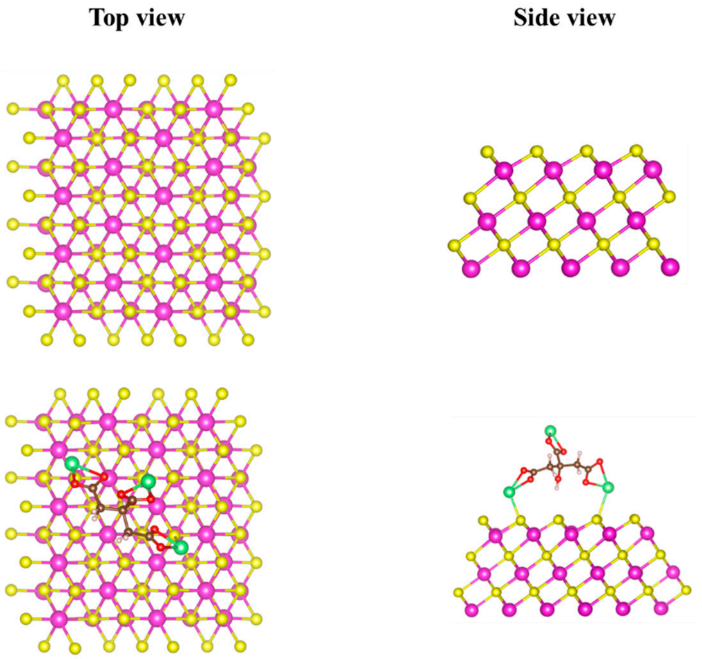

Figure S5. DFT-calculated configuration of CdS (1-10) surface and the adsorption configuration of sodium citrate on CdS (1-10) surface.

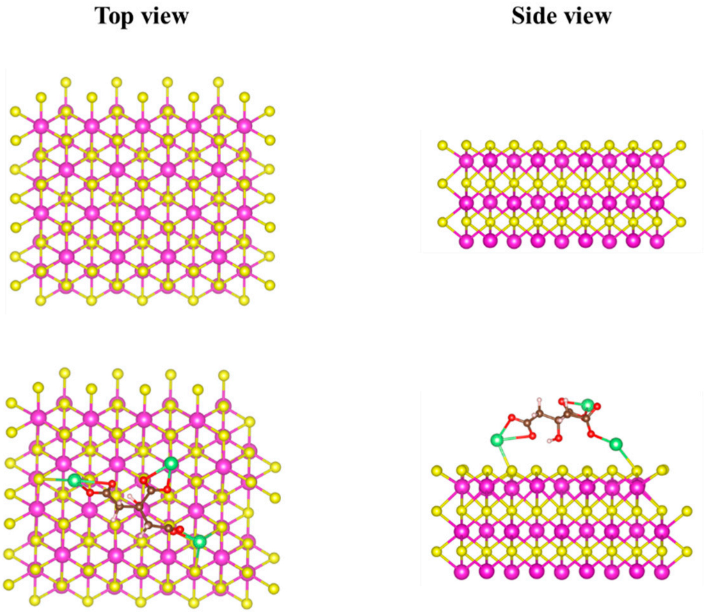

Figure S6. DFT-calculated configuration of CdS (011) surface and the adsorption configuration of sodium citrate on CdS (011) surface.

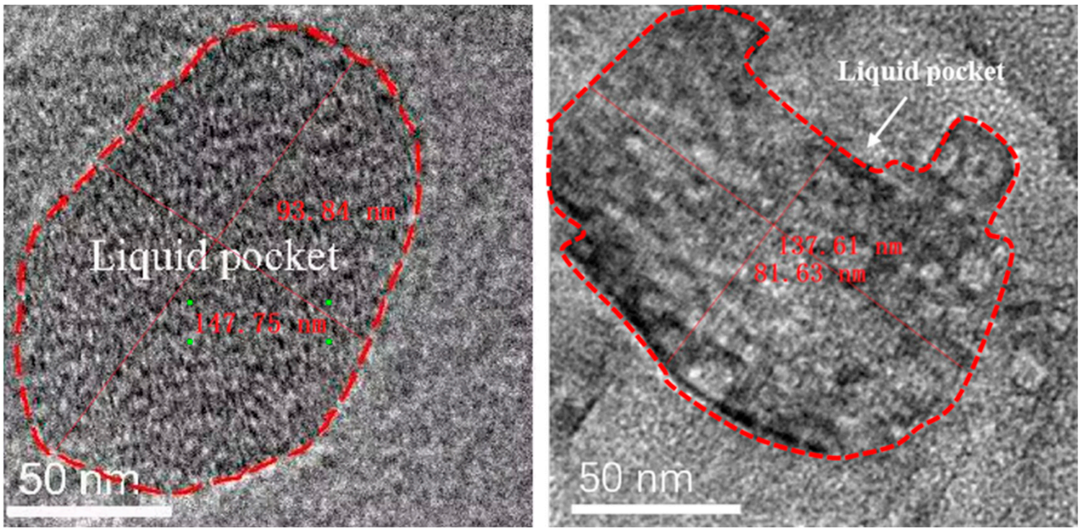

Figure S7. TEM images of liquid pockets.

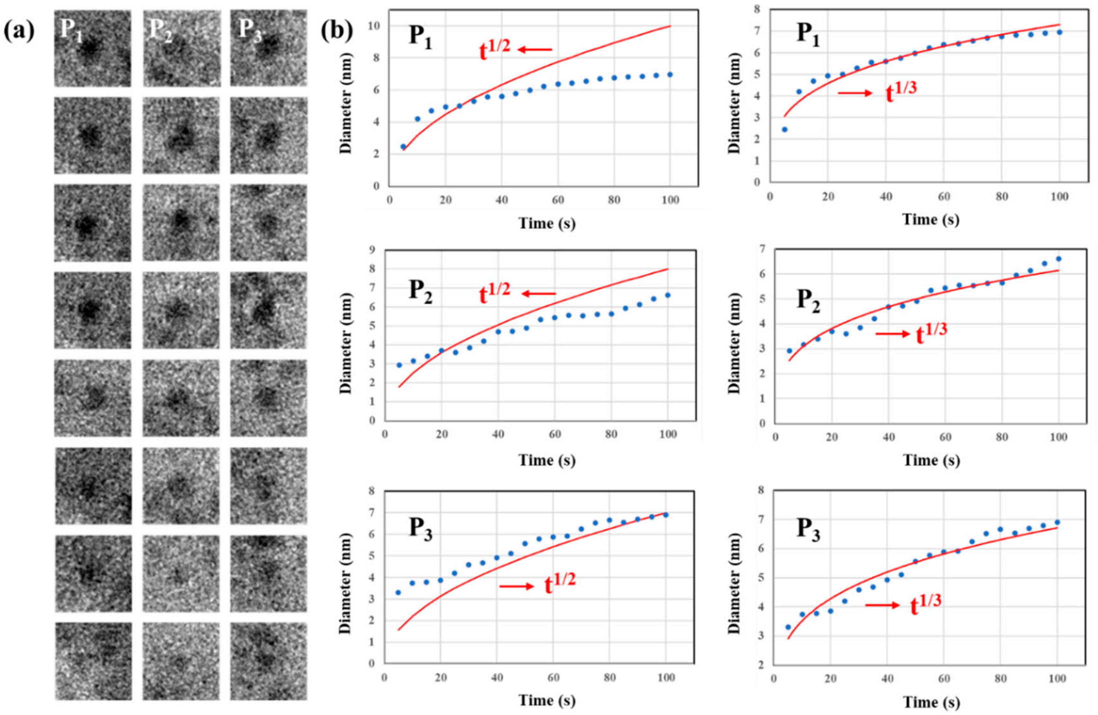

Figure S8. (a) A series of TEM snapshots tracked the growth of three individual nanoparticles labeled as P_1-P_3 in solution. (b) The change in the effective diameter of the CdS nanocrystal.
